# Urethral Hair Bezoar Management With Transcutaneous Neodymium-Doped Yttrium Aluminum Garnet Laser Post-hypospadias Repair: A Clinical Case

**DOI:** 10.7759/cureus.45478

**Published:** 2023-09-18

**Authors:** Aris Kaltsas, Dung Mai Ba Tien, Atsushi Takenaka, Nikolaos Sofikitis, Athanasios Zachariou

**Affiliations:** 1 Department of Urology, University of Ioannina, Ioannina, GRC; 2 Department of Andrology, Binh Dan Hospital, Ho Chi Minh City, VNM; 3 Division of Urology, Department of Surgery, School of Medicine, Faculty of Medicine, Tottori University, Yonago, JPN

**Keywords:** urinary tract infections, neodymium-doped yttrium aluminum garnet laser, transcutaneous, urethral diverticulum, hypospadias repair, urethral hair bezoar

## Abstract

Post-hypospadias repair, hair growth within the urethra, and subsequent hair bezoar formation can lead to significant complications, including urinary tract infections (UTIs) and urinary flow obstruction. Using hair-bearing skin in hypospadias repair can cause these complications. We report a 55-year-old male who underwent two-stage penile hypospadias repair at age three, presenting with recurrent UTIs and lower urinary tract obstruction symptoms. Urethroscopy identified a hair bezoar in a wide-mouth diverticulum of the penile urethra. Post-extraction of the hair bezoar using a rigid cystoscope, transcutaneous neodymium-doped yttrium aluminum garnet (ND:YAG) laser epilation was employed to ablate urethral diverticular hair follicles. Hair bezoars in the urethra, although rare in modern practice, may obstruct urine flow and act as a nidus for UTIs. Transcutaneous ND:YAG laser has emerged as a minimally invasive technique, offering a simple, effective solution for urethral hair removal with minimal complications. Transcutaneous ND:YAG laser epilation serves as a viable first-line treatment for urethral hair follicles following hypospadias repair, emphasizing its significance in preventing recurrent complications in such patients.

## Introduction

Hypospadias is a congenital malformation of the male urethra where the urethral meatus (opening) is located on the ventral side (underside) of the penis rather than at its tip. This condition can range in severity, with the urethral opening being located anywhere from just beneath the tip of the penis (coronal) to the scrotum (penoscrotal) [1]. The exact etiology of hypospadias remains unclear, but it is believed to result from an interruption in the normal development of the urethra during fetal growth. Factors such as genetics, maternal age, and environmental exposures during pregnancy have been suggested as potential contributors [1].

Hypospadias can lead to functional problems, including difficulties in directing the urinary stream and challenges with sexual function later in life [2]. Surgical correction, typically performed in early childhood, aims to relocate the urethral meatus to its typical position and achieve a more conventional penile appearance. However, post-surgical complications can arise, one of which is the growth of hair within the urethra when hair-bearing skin is used in the repair [3].

When hypospadias management includes hair-bearing skin, the hairy urethra and hair bezoars are potential consequences of flap surgical operations. Older patients who underwent multiple staged procedures have been known to present with hairy urethras, with an incidence rate of 6.6% [4]. The inner prepuce, which most closely resembles the urethra, is the best option for treating this issue. Alternative solutions include the bladder mucosa, buccal mucosa, and non-hair-bearing skin of the inner arm or upper thigh. Treatment options for a hairy urethra and hair bezoars are the avoidance of hair-bearing skin use, chemical epilation [5], electro-epilation [6], laser [7], and replacement urethroplasty [8]. This case report describes forceps hair bezoar extraction and transcutaneous neodymium-doped yttrium aluminum garnet (ND:YAG) laser epilation of urethral diverticular hair follicles following hypospadias repair.

## Case presentation

**Figure 1:**
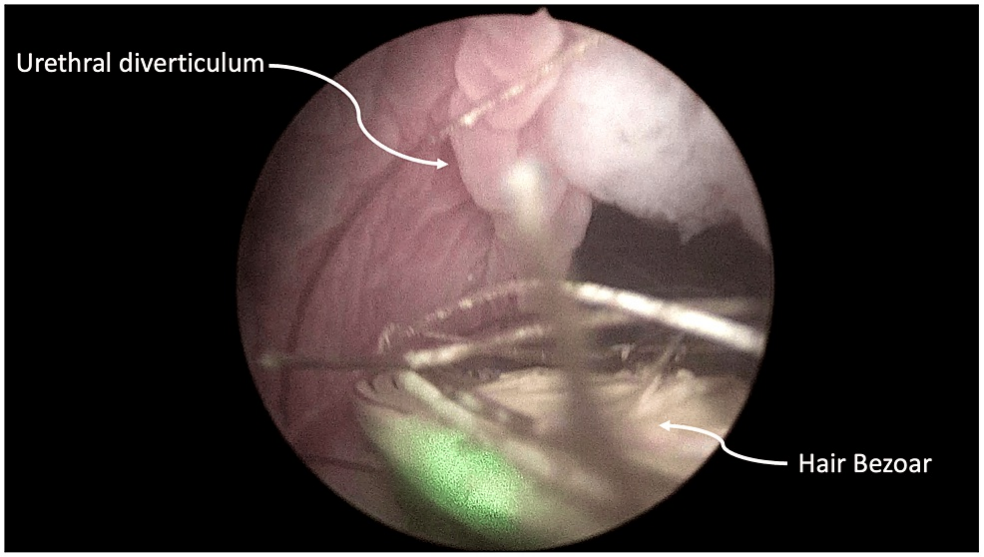
Urethroscopic view of hair bezoar within the urethral diverticulum. Urethroscopic view of the wide-mouth diverticulum in the penile urethra, showing a hair bezoar protruding into the urethral lumen. The bezoar's dense and intertwined hair strands are visible, exemplifying the potential for urinary flow obstruction and nidus for infections.

A 55-year-old male presented with recurrent urinary tract infections five years ago and reported *Escherichia coli* acute prostatitis in the past 12 months. At the age of three, he underwent a two-stage penile hypospadias repair. The patient was complaining of dysuria, urinary frequency, and terminal dribbling. An abdominal-pelvic ultrasound revealed benign prostate hyperplasia with a prostate volume of 39 ml and a prostate-specific antigen (PSA) level of 3 ng/ml. Uroflowmetry presented a plateau-shaped flow pattern with an 8 ml/s maximal flow rate, and there was no post-void residual (PVR) urine in the bladder. Urethroscopy revealed a wide-mouth diverticulum in the penile urethra. It contained a hair bezoar protruding into the urethra lumen (Figure 1). 

A mild stricture, proximal to a urethral diverticulum, obstructed the urethral lumen. A flexible 16 French (Fr) gauge cystoscope passed through the stenosis with moderate pressure. Other pathological findings from the bladder, prostate, and bladder neck were absent.

A guide wire was initially inserted in the urethra under spinal anesthesia with the patient in the dorsal lithotomy position. A dilation of the stricture using coaxial urethral dilators permitted a better visualization of the diverticulum. A rigid cystoscope (19 Fr) with two-prong forceps allowed the extraction of the hair bezoar and all possible residual hair. Marks in the external skin of the penis defined the proximal and distal edges of the diverticulum as a guide for the laser application. A urethral catheter was placed to facilitate the procedure. 

The hair follicles were ablated transcutaneously with a 1064 nanometer (nm) ND:YAG laser. The laser was administered vertically to the skin on the ventral shaft of the penis. The applied skin marks showed the 5 cm^2^ treated surface that matched the urethral hair growth area. There were 50 pulses delivered in all, and specific laser parameters were fluence of 54 J/cm^2^ and 12 mm spot. The catheter was removed after the procedure, and the patient received nonsteroidal anti-inflammatory drugs. It's important to note that we did not prescribe antibiotics post-procedure.

**Figure 2:**
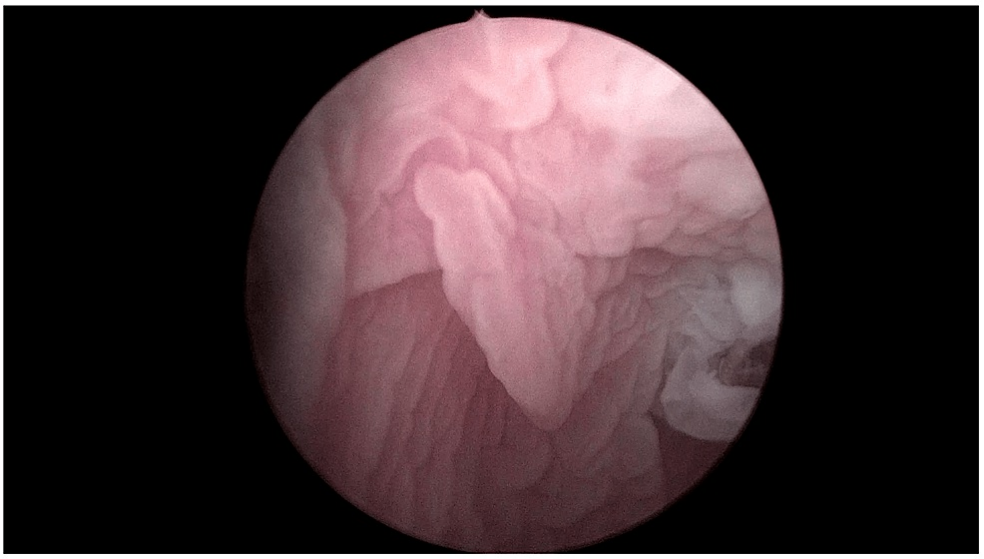
Urethral lumen free of hair, 12 months after initial treatment.

A diagnostic cystoscopy was performed two months later to verify the outcome, and a laser epilation procedure was repeated under local anesthetic to remove the last few remaining hairs. The patient presented an uneventful recovery period but lost the follow-up examinations. He was re-evaluated two months ago with abdominal-pelvic ultrasound, uroflow, and cystoscopy, confirming no recurrence of the hairy urethra and presenting a moderate urethral stricture. Figure 2 illustrates the urethral lumen, which is notably free of hair, captured 12 months after the initial treatment.

## Discussion

Hair growth and urethral diverticula are long-term complications of hypospadias repair and urethroplasty. Fortunately, thanks to preventive strategies, such as the careful selection of non-hair-bearing grafts or flaps, meticulous surgical techniques to avoid diverticula formation, and post-operative care protocols, the incidence of hair bezoars in the urethra has decreased, making it a relatively rare phenomenon in older patients nowadays [9]. Urethral hair or hair bezoars may be a nidus for urinary tract infections (UTIs) and may obstruct urine flow. When treatment is required, hair bezoars are usually managed with carbon dioxide (CO_2_) laser desiccation [10], ND:YAG laser photocoagulation [5], transcutaneous laser ablation [11], diode laser [12], grasper extraction, selective thermocoagulation [6], chemical depilation [5], and open surgical modification [13]. Intraurethral laser application is a minimally invasive technique with minor short-term or long-term complications. Hair regrowth, urinary tract infections, stenosis, and urethra-cutaneous fistula are the most common issues.

A few cases of hair bezoars forming inside the urethral diverticulum, either alone or in conjunction with stones, have been published. These patients underwent open surgical excision [8,14], transcutaneous [11], intraurethral laser hair ablation [9,15,16], or multiple procedures. Some patients have presented with solitary or multiple stones in the neo-urethra associated with hair growth, which can exacerbate complications [8,15,16]. There may be a causal association between stenosis, diverticula, hair growth, and stone formation. Strictures and hair bezoars in the urethra cause urine obstruction, resulting in residual urine in the urethra and gradually creating a diverticulum. The calcium oxalate deposits around the hair and slowly causes a stone to mix with the hair, further enlarging the diverticulum [15]. 

Our patient presented due to recurrent UTIs and lower urinary tract obstruction symptoms. On initial workup, his uroflow chart represented a plateau-shaped flow pattern, indicating possible urethral stenosis, but post-void residual (PVR) was negligible. His main issue was recurrent UTIs, mainly from the urethral hair. 

Finkelstein and colleagues first reported using a transurethral ND:YAG laser for urethral epilation in four patients [7]. They determined that laser power and depth of penetration were significant concerns because the neourethra is delicate and close to the skin, although they reported no complications. The efficacy of the laser procedure relies on the hair color, diameter, and hair bulb depth. Dark hair (eumelanic) absorbs a substantial portion of the laser energy. The temperature rise in the hair bulb and bulge damages the follicle, resulting in total hair destruction. 

Crain et al. were the first to report on percutaneous laser hair ablation [11]. The closeness of the hair bulb to the skin and the thickness of the hair were enough to counteract any potential drawback. One of the significant advantages of the procedure is the use of topical or no anesthesia. We opted for the transcutaneous ND:YAG laser for similar reasons, given its simplicity, safety, and established efficacy in soft tissue applications.

## Conclusions

Transcutaneous ND:YAG laser epilation offers a minimally invasive method for treating urethral hair follicles following hypospadias correction. This method can be safely employed as a first-line surgical choice in such patient populations.
